# Advances in Biomaterials for Tissue Regeneration: From Scaffold Design to CAP-Enabled Interfaces and AI-Driven Optimization

**DOI:** 10.3390/biomimetics11050330

**Published:** 2026-05-09

**Authors:** Laura Del Gaudio, Stefano Lattanzio, Roberta Di Pietro, Silvia Sancilio

**Affiliations:** Department of Medicine and Ageing Sciences, University “G. d’Annunzio” Chieti-Pescara, via Dei Vestini 31, 66100 Chieti, Italy; laura.delgaudio@phd.unich.it (L.D.G.); r.dipietro@unich.it (R.D.P.); s.sancilio@unich.it (S.S.)

**Keywords:** biomaterials, tissue engineering, extracellular matrix, 2D materials, 3D printing, cold atmospheric plasma, artificial intelligence

## Abstract

Biomaterials play a central role in tissue engineering and regeneration by providing scaffolds that support cell adhesion, proliferation and differentiation while modulating the surrounding microenvironment. They represent promising alternatives to traditional surgical approaches that may lead to complications or tissue damage, and their performance is influenced by chemical composition, mechanical behavior, architecture and interfacial properties, all of which can be precisely tuned through advanced fabrication and surface modification strategies. This review synthesizes evidence from a comprehensive literature search across major scientific databases, focusing on highly cited studies and available clinical data, and examines natural and synthetic biomaterials, their biological responses, functional characteristics, and surface modification methods. Emphasis is placed on Cold Atmospheric Plasma (CAP), which selectively modifies the outermost nanolayer of materials, enhancing hydrophilicity, functional group density, protein adsorption and overall cell–material interactions, as well as improving drug loading capacity. The review also considers stem cell interactions with biomaterials and emerging applications of artificial intelligence (AI) for predicting performance and guiding material optimization. Overall, the analysis highlights that natural matrices provide intrinsic bioactivity, synthetic polymers offer tunable mechanics and degradation profiles, and composite systems integrate these advantages. Advances in technologies such as electrospinning and 3D/4D printing enable precise control over architecture, supporting cell colonization and vascularization. Collectively, developments in CAP treatments and AI-driven design strategies are strengthening the regenerative potential of biomaterials and advancing their clinical translation.

## 1. Introduction

Surgical procedures remain a cornerstone of clinical practice; however, their widespread use has contributed to an increased incidence of iatrogenic injuries and postoperative complications. Tissue engineering (TE) and tissue regeneration (TR) offer promising alternatives, aiming to restore damaged tissues through the integration of biomaterials, cells, and biochemical cues [1,2,3].

In recent years, interest in TE has grown substantially due to its potential to address irreversible tissue damage and chronic diseases. This approach relies on scaffolds that recreate an instructive microenvironment, supporting cell proliferation, differentiation, and matrix deposition while degrading in a controlled manner in parallel with tissue formation [3,4]. TE is inherently multidisciplinary, integrating engineering, biology, medicine, and materials science to design biocompatible materials tailored to specific clinical needs [1]. Applications span orthopedics, cardiovascular repair, dental regeneration, skin substitutes, and controlled drug delivery systems. Despite significant progress, key challenges remain in optimizing scaffolds for complex tissues and refining fabrication techniques [2].

In this review, we

(1) Compare natural and synthetic biomaterials, including hybrid systems;
(2) Outline advanced fabrication strategies (electrospinning and 3D/4D printing) and architectural parameters;
(3) Highlight surface engineering, with emphasis on CAP;
(4) Discuss cell–material crosstalk and immunomodulation;
(5) Explore the role of AI in predictive design;
(6) Address safety considerations and translational challenges.

Unlike existing reviews that treat biomaterials, plasma technologies, or artificial intelligence as separate domains, this work proposes an integrated framework in which scaffold composition, CAP-enabled surface engineering, and AI-driven optimization are considered interdependent elements of next-generation tissue regeneration strategies.

## 2. Materials and Methods

A narrative review was conducted using PubMed, Google Scholar, and ScienceDirect as research databases. No temporal restrictions were applied to the literature search. The main keywords included “Tissue engineering,” “Tissue regeneration,” “Biomaterials,” “Scaffold”, “Bioscaffold”, “Electrospinning”, “3D printing”, “4D printing”, “Cold Atmospheric Plasma”, “Artificial Intelligence”, and “Cells”. Only articles published in English were considered. Studies were selected and prioritized based on relevance, methodological rigor, and citation impact.

## 3. Results and Discussion

The findings from the selected studies are presented and compared below, followed by an integrated discussion of their implications for tissue regeneration strategies.

### 3.1. Tissue Engineering and Scaffold Categories

TE integrates engineering principles with biological systems to restore or enhance the function of damaged tissues [1,2]. Central to this approach is the creation of a microenvironment that regulates cell proliferation, differentiation, and migration through biochemical and mechanical cues [3,4]. Two principal strategies can be distinguished: scaffold-based and scaffold-free approaches.

Scaffold-based TE relies on temporary three-dimensional (3D) structures designed to mimic the organization of the extracellular matrix (ECM) and support spatial cell arrangement, thereby promoting adhesion, viability, and lineage commitment. The ECM itself is a highly organized, 3D macromolecular network composed of structural proteins, glycoproteins, and proteoglycans which regulate cellular behavior through both biochemical cues and biomechanical signaling [5,6]. Accordingly, scaffold properties such as architecture, stiffness, and surface chemistry play a critical role in directing cell fate.

In contrast, scaffold-free approaches exploit the intrinsic ability of cells to self-organize into functional tissues constructs, either in the presence of supportive substrates (e.g., Matrigel™ or acellular matrices) or through direct implantation [7,8].

Additional strategies include the use of decellularized ECM as a biologically derived scaffold that preserves native biochemical composition and structural architecture while supporting tissue-specific cell populations [9,10,11]. Overall, the success of TE depends on the identification of appropriate cell sources, the selection or design of suitable biomaterials, and the engineering of microenvironments that recapitulate physiological tissue development. Despite significant advantages, both scaffold-based and scaffold-free approaches present important limitations. Scaffold-based strategies are often hindered by inadequate vascularization, incomplete replication of native tissue complexity, and limited long-term integration, particularly in thick or highly metabolic tissues [2,3,12,13]. Conversely, scaffold-free strategies, while biologically appealing due to their reliance on intrinsic cell self-organization, are frequently constrained by poor mechanical stability, limited scalability, and challenges in manufacturing and reproducibility [7,8]. These limitations highlight a critical need for integrated approaches combining rational biomaterial design, surface functionalization, and advanced fabrication technologies to bridge biological relevance with clinical applicability.

### 3.2. Cells Involved in Tissue Engineering

Multiple stem and progenitor cell sources, including embryonic stem cells (ESCs), induced pluripotent stem cells (iPSCs), and adipose-derived mesenchymal stem cells (ADMSCs), have demonstrated promising outcomes in various tissue engineering (TE) applications involving renal, tracheal, cartilage, and ureteral models [14,15,16,17,18]. These findings highlight the critical influence of the cellular microenvironment while also underscoring the need to better understand long-term cell fate and functional stability, particularly blood-contacting or regenerating devices. Among available stem cell types, ESCs and iPSCs are especially versatile due to their pluripotency and broad differentiation capacity. In addition, stem cells derived from perinatal tissues such as the umbilical cord, amniotic fluid, and placenta [14,15,16,17,18], represent ethically accessible and immunologically favorable alternatives.

Several studies have investigated the ability of stem cells to reconstruct functional renal and epithelial structures (Table 1). Lanza et al. successfully cloned renal cells from bovine fetuses and reimplanted them in vivo, where they self-organized into tissue-like structures capable of waste filtration [19]. Ross et al. seeded murine ESCs into decellularized rat kidney extracellular matrix, achieving both proliferation and lineage-specific differentiation [20]. Goto et al. further demonstrated that the amniotic membrane can serve as an effective biological scaffold for epithelial and fibroblast seeding, leading to the formation of a multilayered tracheal epithelium without the addition of exogenous growth factors [21]. Collectively, these studies demonstrate the potential of stem cell-based approaches, while also highlighting the need for deeper investigation into their long-term fate and integration dynamics in vivo.

Beyond stem cell-based strategies, differentiated autologous cells are already used in clinically approved TE applications. A notable example is Matrix-Induced Autologous Chondrocyte Implantation (MACI), an FDA-approved technique in which autologous chondrocytes are seeded onto a porcine collagen membrane for the repair of focal cartilage defects [22]. Dermal fibroblasts are likewise widely employed in wound-healing constructs and skin substitutes [20,23,24].

Adipose-derived stem cells (ADSCs) have attracted increasing interest due to their accessibility, abundance, and minimally invasive harvesting. Importantly, ADSCs exhibit strong angiogenic potential, supporting neovascularization, an essential process for tissue repair and regeneration [25]. Zhao et al. successfully differentiated ADSCs into smooth muscle and urothelial phenotypes in vitro, subsequently implanting them in a rabbit ureteral model. After eight weeks, the engineered constructs exhibited organized ureter-like structures without signs of inflammation, demonstrating the feasibility of ADSC-based ureteral regeneration [26].

Overall, although cell-based strategies demonstrate significant regenerative potential, their efficacy remains highly dependent on the surrounding biomaterial context. Variability related to cell source, donor-specific factors, differentiation state, and long-term phenotypic stability continues to compromise reproducibility and translational reliability, particularly in complex or clinically demanding applications [14,15,16,17,18,25,26]. These limitations further underscore the pivotal role of biomaterial design in directing, supporting, and stabilizing cellular behavior.

**Table 1:** Studies about cells in TE.

Study	Type of Cells	Experimental Model	Main Findings
Lanza et al. [19]	Renal cells cloned from bovine fetuses	In vitro cultivation and in vivo reimplantation	Cells self-organized into structures capable of waste product elimination
Ross et al. [20]	Murine embryonic stem cells	Seeding onto rat kidney extracellular matrix	Cells proliferated and differentiated
Goto et al. [21]	Epithelial cells and fibroblasts	Amniotic membrane used as basement membrane	Formation of a multilayered epithelium without the need for added growth factors
Biniazan et al. [25]	Adipose tissue stem cells	In vitro cultivation	Formation of new blood vessels
Zhao et al. [26]	Adipose tissue stem cells from kidney	In vivo cultivation (rabbit model)	Formation of ureter structure: no signs of inflammation are observed

In vitro: experiments conducted in controlled laboratory conditions outside a living organism. In vivo: experiments performed within a living organism. ECM: extracellular matrix used as a structural scaffold for cell seeding. The amniotic membrane acted as a natural basement membrane supporting epithelial stratification. No exogenous growth factors were added in Goto et al. [21]. The absence of inflammation reported in Zhao et al. [26] refers to histological evaluation after implantation.

### 3.3. Biomaterials for TE: Natural, Synthetic, and Hybrid Strategies

Biomaterials are engineered to interact safely and effectively with human tissues, providing structural and biochemical support essential for tissue regeneration. Their properties (including chemical composition, mechanics, surface topography, and degradation kinetics) must be carefully tuned to recreate a microenvironment that promotes cell adhesion, proliferation, differentiation, and matrix deposition, both in vitro and in vivo [3,27,28]. Advanced fabrication technologies, such as electrospinning and 3D printing, now enable precise control over scaffold architecture at the nano- and microscale, allowing the modulation of pore geometry and surface features that guide cellular behavior. Rather than relying on high concentrations of costly growth factors, several strategies enhance biomaterial performance through the incorporation of ECM-derived proteins such as collagen, elastin, gelatin, and fibronectin, which improve bioactivity and tissue-specific responses [29,30,31].

Surface chemical properties, including charge, functional groups, and wettability, represent key regulators of protein adsorption and subsequent cell adhesion. In particular, hydrophilicity strongly influences the adsorption and conformation of serum proteins, which in turn modulate cell–material interactions [32,33].

Scaffolds can be fabricated either from synthetic polymers, such as PEG, PLGA, PCL, or from naturally derived ECM components, including collagen, laminin, cellulose, and chitosan (Table 2). Both classes are widely used in TE: natural biomaterials provide intrinsic bioactivity and structural similarity to native ECM, whereas synthetic polymers offer tunable mechanical and degradation properties. Increasingly, hybrid systems combining natural and synthetic components are employed to synergistically integrate the advantages of both.

**Table 2:** Classification of biomaterials.

Category	Subcategory	Examples
Natural	Biopolymers	Polylactic acid (PLA) and polyhydroxyalkanoates (PHAs)
	Polysaccharides	Hyaluronic acid (HA), alginate, cellulose, and chitosan
	Polypeptides and proteins	Collagen, gelatine, and fibroin
Synthetic	PCL	Polycaprolactone
	PEG	Polyethylene glycol
	PGA	Polyglycolic acid
	PLGA	Polylactic glycolic acid

Synthetic biomaterials inherently lack biological recognition motifs found in native extracellular matrix components; however, advanced design strategies enable them to precisely modulate cell behavior through controlled chemical composition, tunable degradation profiles, surface topography, and biofunctionalization with bioactive cues [29,34,35].

From a comparative perspective, natural, synthetic, and hybrid biomaterials exhibit complementary advantages and limitations (Table 3). Natural biomaterials intrinsically recapitulate the biochemical cues of the ECM, thereby promoting cell adhesion and lineage commitment; however, they often present limited mechanical strength, batch-to-batch variability, and reduced scalability. In contrast, synthetic polymers provide reproducible mechanical properties, tunable degradations kinetics, and scalable manufacturing, but lack intrinsic bioactivity and typically require surface functionalization. Hybrid biomaterials aim to integrate these features by combining bioactive natural components with mechanically robust synthetic matrices, enabling tissue-specific optimization of both biological and structural performance [22,29,36,37].

**Table 3:** Comparative features of biomaterial classes.

Feature	Natural	Synthetic	Hybrid
Bioactivity	High	Low	High
Mechanical strength	Low–moderate	High	Tunable
Degradation control	Limited	Precise	Tunable
Reproducibility	Low	High	Moderate
Clinical scalability	Moderate	High	Emerging

Comparative overview of major biomaterial classes based on reported trends in tissue engineering literature [29,34,36,37].

From a critical perspective, no single class of biomaterials can be considered universally superior. Natural biomaterials intrinsically recapitulate extracellular matrix cues and promote favorable cell–material interactions, yet they are often limited by batch-to-batch variability, reduced mechanical strength, and challenging standardization [36,37,38]. Synthetic polymers, by contrast, offer highly controlled mechanical properties, degradation kinetics, and reproducibility but generally lack biological recognition signals, requiring additional surface modification or biofunctionalization [29,34,35]. Hybrid biomaterials aim to reconcile these opposing features; however, their increased compositional and processing complexity introduces challenges related to manufacturing scalability, regulatory approval, and long-term performance predictability.

Addressing these trade-offs remains a key unresolved challenge in the development of clinically translatable biomaterial platforms.

#### 3.3.1. Natural Biomaterials

Among biomaterials, those of biological origin have been extensively used in recent years for the fabrication of bioscaffolds. Owing to their intrinsic bioactivity, they can effectively mimic the composition, structure, and functional properties of the ECM [36,37,39]. Natural biomaterials are derived from renewable sources, including plants, animals and microorganisms, and exhibit physiological relevant characteristics that enhance cellular compatibility and adaptability [22,37,40]. They are generally considered biocompatible, with low toxicity, non-genotoxicity, and non-teratogenicity profiles.

The main classes of natural biomaterials used in TE and regenerative medicine include

- Bio polyesters, such as PLA, PHAs and their derivatives;
- Polysaccharides, such as HA, alginate, cellulose and chitosan.;
- Polypeptides and proteins, such as collagen, gelatin, fibroin, polyglutamic acids and antimicrobial peptides (AMPs) which contribute to ECM-like biofunctionality.

#### 3.3.2. Collagen and Cellulose

Collagen represents the most abundant natural biomaterial employed in tissue engineering (TE) [40], owing to its widespread presence in the extracellular matrix (ECM), where it accounts for approximately 85% of its dry weight [41]. Its excellent biocompatibility and intrinsic ability to support cell adhesion and proliferation have led to its extensive application in the regeneration of a broad range of tissues, including skin, bone, cartilage, tendons, skeletal muscle, bladder, vascular structures, spinal cord, neural tissue, teeth, periodontium, and cornea [40]. Cellulose, another naturally derived biomaterial, has gained increasing attention for its ability to enhance and complement the properties of traditional biomaterials. In particular, it contributes to improved mechanical stability and promotes cell proliferation [42]. Additionally, its highly porous structure plays a key role in enabling the sustained release of drugs, growth factors, and other bioactive molecules, making it especially valuable in controlled delivery systems [43].

Despite these advantages, both collagen and cellulose exhibit inherent limitations, including relatively low mechanical strength, rapid degradation rates, and batch-to-batch variability. These drawbacks restrict their use as standalone materials in load-bearing or long-term applications. Consequently, their combination with synthetic polymers or other reinforcing components is often required to achieve the mechanical robustness and structural reliability necessary for advanced tissue engineering applications [34,36,37,42].

#### 3.3.3. Decellularized Extracellular Matrix

ECM-derived scaffolds are generated through decellularization processes that remove cellular components while preserving biochemical cues, structural organization, and mechanical properties [38]. Common decellularization techniques include treatment with non-ionic or zwitterionic detergents, enzymatic digestion (e.g., trypsin), and solvent-based extraction using alcohols [38,44,45]. To preserve bioactive components, ECM scaffolds are often processed in a dehydratated state, tipically via lyophilization or vacuum pressing [45,46,47]. Depending on the processing method, ECM-derived scaffolds can be fabricated in various formats, including single-layer sheets [47,48], multilayer constructs [45,48], powders [45,49], hydrogels [22,50], and hybrid systems [22,37,51]. The choice of fabrication strategy should be guided by the intended clinical application. Following implantation, ECM scaffold degradation is initiated as part of the host response, triggering a cascade of events that promotes tissue repair [43,45]. This process involves the recruitment of stem/progenitor cells (SCs), the activation of the innate immune system, and subsequent cell proliferation, ultimately leading to constructive tissue remodeling [38,45,52]. ECM degradation results in the release of structural proteins and bioactive fragments, including collagens, elastin, fibronectin, laminins, proteoglycans, and glycoproteins, which constitute the core matrisome and regulate cell behavior during regeneration [53]. In addition, ECM breakdown generates cryptic peptides with regulatory functions that further influence cell recruitment and differentiation. ECM scaffolds also contain matrix-bound nanovesicles (MBVs), which deliver bioactive lipids, proteins, and microRNAs (miRNAs), thereby modulating immune responses and supporting stem/progenitor cell recruitment [52]. Growth factors embedded within the matrix, such as fibroblast growth factor (FGF), vascular endothelial growth factor (VEGF), platelet-derived growth factor (PDGF), and transforming growth factor-beta (TGF-β), are also exposed or released during scaffold remodeling and play a key role in sustaining regenerative signaling [45]. At the intracellular level, ECM-mediated mechanical cues are trasmitted through integrin-dependent focal adhesion complexes and downstream effectors, including focal adhesion kinase (FAK) and proto-oncogene tyrosine-protein kinase (Src), which ultimately regulate gene expression and cell fate decisions via mechanotransduction pathways [54]. Together, these biochemical and biomechanical signals orchestrate the transition from inflammation to proliferation and, ultimately, to constructive tissue remodeling [55].

Despite these advances, the mechanisms underlying stem/progenitor cell recruitment by ECM scaffolds remain only partially understood.

ECM-derived scaffolds are widely regarded as highly effective due to their ability to recreate a phisiologically relevant microenvironment that supports both mechanical integrity and cellular differentiation. Their performance, however, depends on multiple factors, including tissue source, decellularization protocol, host immune response, and patient-specific variables such as age and pathological condition.

Neverthless, ECM-based scaffolds present several limitations, including donor variability, incomplete decellularization, potential immunogenic residues, limited mechanical strength, and challenges in standardization and large-scale production [38,44,45,55].

In recent years, increasing attention has been directed toward the development of more cost-effective and environmentally sustainable decellularization strategies. Compared with conventional detergent-based protocols, physical methods such as freeze–thaw cycles, agitation, and osmotic shock reduce chemical usage and processing costs. Enzymatic treatments (e.g., trypsin or nucleases) can also be applied at low concentrations to minimize environmental impact. Furthermore, the use of non-ionic detergents and aqueous-based systems has emerged as a more sustainable alternative, aiming to balance effective decellularization with preservation of ECM ultrastructure and bioactivity. Although these approaches may show reduced efficiency in dense tissues, they represent promising strategies for scalable and eco-conscious ECM production when appropriately optimized [38,44,45,47].

#### 3.3.4. Synthetic Biomaterials

Synthetic biomaterials are composed of engineered polymers, including PLA/PLLA/PLCL, PCL, PGA, PLGA, and PEG, and offer controlled mechanical behavior, high reproducibility, and tunable degradation kinetics [34,35]. Although they lack intrinsic bioactivity, their surfaces can be readily functionalized or combined with bioactive agents to enanches cell–material interactions. These include growth factors (such as FGF, VEGF, PDGF, and TGF-β, which promote cell proliferation, angiogenesis, and tissue regeneration) [45], bioactive peptides (e.g., ECM-derived peptides that enhance integrin-mediated cell adhesion) [53], small bioactive molecules (including quercetin, κ-carrageenan, dopamine, sericin, and other plant- or animal-derived compounds with pro-regenerative or immunomodulatory properties), extracellular vesicles such as EVs and MBVs which deliver regulatory proteins, lipids, and microRNAs involved in cell signaling and immune modulation [52], and cytokines that modulate inflammation and promote a pro-healing microenvironment. This versatility makes synthetic polymers well suited for fabrication of scaffolds, drug delivery systems, and implantable devices [56].

Despite their widespread use and tunability, synthetic biomaterials continue to face challenges related to limited biological signaling and insufficent dynamic reciprocity with surrounding tissues. While surface functionalization and the incorporation of bioactive components can significantly enhance performance, issues such as long-term stability, immune modulation, and predictive control of biological responses remain to be fully addressed [29,34,56].

#### 3.3.5. Hybrid Composites

Hybrid biomaterials combine the structural reliability of synthetic polymers with the bioactivity of natural components. Representative examples include PCL/cellulose nanofibers, PCL combined with ECM, and PLA or PCL reinforced with hydroxyapatite or graphene oxide [57,58]. These systems enanche mechanical performance, osteoconductivity, and cellular response. For instance, PCL scaffolds reinforced with cellulose nanofibers exhibit improved mechanical strength and increased cell proliferation compared with pure PCL [59]. Similarly, ECM-enriched PCL scaffolds retain the mechanical stability of synthetic matrices while promoting enhanced cell adhesion and proliferation [51].

Hybrid systems can also incorporate nanoparticles, growth factors, or bioactive peptides to modulate signaling pathways relevant to bone, cartilage, or soft tissue regeneration. Surface modification techniques, including plasma treatment or peptide grafting, further improve bioactivity and cellular integration [34,60,61].

Overall, natural biomaterials are primarily associated with intrinsic bioactivity, whereas synthetic materials offer precise control over mechanical and degradation properties; hybrid systems provide a rationally engineered balance between these features. The selection of an appropriate platform ultimately depends on the mechanical requirements of the target tissue, the desired remodeling timeline, and the specific immune context.

### 3.4. Advanced Fabrication: From Electrospinning to 3D/4D Printing

Electrospinning enables the fabrication of nano- and micro-fibrous scaffolds with controllable fiber alignment and high surface-to-volume ratios, features that regulate cell adhesion, migration, and differentiation [6,29,30]. Advanced approaches, such as co-electrospinning and post-functionalization, allow the incorporation of biochemical gradients and development of multiphase architectures.

3D bioprinting allows the precise fabrication of customized scaffolds using bioinks composed of hydrogels and living cells. These constructs facilitate cell infiltration, nutrient diffusion, and tissue-specific maturation, with porosity and geometry finely tunable through digital design [37]. However, 3D-printed materials are inherently static. To overcome this limitation, four-dimensional (4D) printing introduces dynamic constructs capable of responding to external stimuli, such as temperature, pH, light, magnetic or electrical fields [62]. These stimulus-responsive materials can adapt their structure or properties over time, thereby functional integration and performance. 4D-printed scaffolds based on biocompatible polyesters such as PLA and PCL show improved cell adhesion, proliferation, and differentiation [63,64]. Nevertheless, architectural parameters (pore size and interconnectivity) remain crucial: excessively small pores can restrict nutrient transport, whereas overly large pores may reduce the available surface area for cell attachment [12]. Optimal pore size ranges are tissue-specific (Table 4) [13,65,66,67,68]. Overall, electrospinning and 3D/4D printing represent powerful and complementary strategies for designing biomaterials with highly controlled architectures tailored to TE applications.

### 3.5. Surface Engineering for Bioactivity and Hemocompatibility

Although bulk material properties determine mechanical behavior and degradation, the surface primarily governs initial protein adsorption, cell adhesion, and hemocompatibility (Table 5). Strategies such as peptide grafting, ECM coatings, antifouling layers, nanoparticle decoration, and plasma treatments enhance bioactivity by modulating surface chemistry, wettability, and nano-topography [32,33].

Recent studies highlight the potential of intelligent scaffolds that respond to biological signals, self-healing hydrogels with ECM-like properties, and novel cell populations (e.g., lipo-chondrocytes) to improve tissue integration and regeneration [23,24,69]. Among surface modification techniques, plasma-mediated grafting offers high stability and enhanced resistance to thrombosis, making it particularly suitable for long-term implants [60]. In addition, plasma treatments enable the introduction of reactive functional groups for anchoring peptides or proteins without altering mechanical properties [22]. Beyond chemistry, surface topography, including roughness, fiber alignment, and anisotropy, plays a crucial role in regulating cell adhesion, migration, and mechanotransduction, ultimately influencing lineage commitment and tissue organization [5,6].

### 3.6. Cold Atmospheric Plasma in Biomaterial Surface Modification

Cold Atmospheric Plasma (CAP) has emerged as a versatile surface engineering strategy to enhance biomaterial–cell interactions. Alongside established approaches such as chemical functionalization, bioactive coatings, and nanoscale topographical modifications, CAP offers the distinct advantage of selectively modifying the surface while preserving bulk properties [22,60,70]. Plasma is a partially ionized gas composed of electrons, ions, radicals, excited species, neutral particles, electromagnetic fields, and UV–Vis radiation. When generated at atmospheric pressure and near room temperature, CAP enables rapid, solvent-free, and energy-efficient surface modification, making it highly attractive for biomedical applications [70]. A key feature of CAP is its surface-selective action: only the outermost nanometric layer is affected, leaving mechanical integrity and thermal stability unchanged. This makes it particularly suitable heat- and solvent-sensitive polymers such as PCL, PLA, and other aliphatic polyesters widely used in TE [71]. Beyond simple activation, CAP induces functionalization, crosslinking, and nanoscale restructuring through the combined action of reactive oxygen and nitrogen species (ROS/RNS), electric fields, UV photons, and ion bombardment (Table 6).

- CAP treatment significantly improves wettability, thereby enhancing protein adsorption and cell adhesion. For instance, in electrospun poly(vinyl alcohol)/poly(L-lactic acid) (PVA/PLLA) nanofibrous scaffolds, CAP reduces the water contact angle (WCA) from approximately 110° to 50°, promoting ECM protein adsorption and improving fibroblast and osteoblast attachment. This effect is accompanied by increased alkaline phosphatase (ALP) activity and accelerated calcium deposition in mesenchymal stem cells (MSCs), indicating enhanced osteogenic differentiation [72,73].
- In addition to supporting adhesion, CAP modulates cell proliferation and differentiation. In PCL/PLGA scaffolds loaded with β carotene, CAP enhances MSC adhesion, proliferation, and mineralization, while upregulating osteogenic markers such as RUNX2, SOX9, and osteonectin [74]. Similar effects have been observed in human calvaria osteoblasts (HCOs), with increased expression of ALP, COL1A1, RUNX2, and BMP2, suggesting activation of maturation [75].
- CAP is also effective in improving the performance of additively manufactured scaffolds. In 3D-printed PLA constructs, it increases nanoscale roughness (from approximately 1.2 nm to over 27 nm) while enhancing surface chemistry and hydrophilicity, resulting in improved osteoblast attachment, spreading, and proliferation [76].
- Beyond bone applications, CAP demonstrates versatility across different tissues. For example, electrospun PCL scaffolds loaded with TGF β1-releasing microspheres exhibit enhanced hydrophilicity, increased vitronectin adsorption, and deeper cell infiltration following CAP treatment. In 3D-cultures, these scaffolds promote glycosaminoglycan (GAG) deposition and type II collagen expression, supporting cartilage tissue maturation [77].
- The biological effects of CAP are primarily driven by ROS/RNS generation, UV radiation, localized electric fields, and reactive radicals. These factors increase surface energy, introduce polar functional groups (hydroxyl, carbonyl, and carboxyl), and modify surface topography. As a result, wettability improves, facilitating selective adsorption of ECM proteins such as fibronectin and vitronectin, activation of integrin-mediated adhesion, and downstream signaling pathways regulating migration, proliferation, and lineage commitment [78,79].

However, when applied to cell-laden systems, CAP parameters must be carefully controlled. Excessive ROS/RNS generation can induce redox imbalance and impair cell viability and function in a dose-dependent manner, especially in 3D constructs [74,75,79]. CAP can also be combined with pre-functionalized scaffolds containing bioactive molecules, nanoparticles, or therapeutic agents, or used as a pre-treatment to enhance interfacial bonding and mechanical stability in composite systems (e.g., PLA/CaCO_3_) [80,81].

**Table 6:** Comparative table of CAP effects on different biomaterials.

Biomaterial/System	Type of CAP Treatment	Physicochemical Modifications	Biological Response	Main Application	
PVA/PLLA	Atmospheric cold plasma; low temperature	WCA reduction from 110° to 50°; increased surface energy	Enhanced fibroblast and osteoblast adhesion; ↑ ALP; ↑ Ca^2+^ deposition; ↑ ECM protein adsorption	Bone/soft tissue regeneration	[72]
PCL/PLGA + β-carotene	Surface activation via CAP	Introduction of polar groups (C–O and C=O); improved wettability	↑ MSC adhesion, proliferation, and mineralization; ↑ osteogenic markers (RUNX2, SOX9, and osteonectin)	Bone regeneration	[74]
Human calvaria osteoblasts (HCOs)	Direct CAP exposure	Controlled ROS generation; sub-nm topography modification	↑ ALP, COL1A1, RUNX2, and BMP2; ↑ mineralization	Osteoinduction/bone models	[75]
PLA	CAP with high reactive species density	Roughness increases from 1.2 to 27.6 nm; ↑ –OH/–COOH; ↑ hydrophilicity	Improved osteoblast adhesion, spreading, and proliferation; better 3D colonization	Bone regeneration	[76]
PCL + TGF-β1 microspheres	CAP pre-treatment	↑ Vitronectin adsorption; ↑ hydrophilicity; improved cell infiltration	↑ GAG; ↑ collagen II; enhanced cartilage maturation	Cartilage regeneration	[77]
PLA/CaCO_3_ composites	CAP interfacial enhancement	Improved polymer–mineral bonding; ↑ hydrophilicity; ↑ mechanical stability	↑ MSC proliferation; improved biocompatibility	Bioactive composites/bone	[80,81]
PCL	CAP surface functionalization	Carbonyl/carboxyl introduction; ↑ surface energy	↑ fibroblast, MSC, and chondrocyte adhesion and proliferation	Soft tissue, cartilage, and nerve	[78,79]

CAP increases (↑) hydrophilicity, activates functional groups, and modifies the design, resulting in improved cell adhesion, proliferation, and differentiation. CAP is a versatile tool for designing next-generation scaffolds for bone, cartilage, and soft tissue regeneration. The symbol ↑ indicates an increase in the corresponding parameter.

Despite its advantages, CAP-based surface modification presents several limitations that must be considered for reliable translation. Plasma effects are highly sensitive to operational parameters such as power, gas composition, exposure time, and electrode–substrate distance, which may affect reproducibility across laboratories. Moreover, plasma-induced functional groups may undergo surface aging, leading to reduced long-term stability unless appropriate storage or stabilization strategies are implemented. Excessive generation of reactive oxygen and nitrogen species (ROS/RNS) may also induce undesirable oxidative effects if not carefully controlled [70,78,79].

### 3.7. Artificial Intelligence as a Driving Force in Next-Generation Biomaterial Design

Artificial intelligence (AI) is rapidly transforming biomaterials science by enabling data-driven, predictive design strategies that extend beyond traditional approaches [82]. AI algorithms can analyze large, multidimensional datasets encompassing polymer chemistry, surface modifications, mechanical properties, degradation kinetics, and biological responses, allowing the prediction of optimal material formulations and processing conditions with high accuracy [83]. This capability is particularly relevant for emerging technologies such as CAP, where plasma–material interactions are highly complex and governed by multiple nonlinear parameters, including gas composition, power, exposure time, distance, and humidity [84]. AI facilitates the rational design of tailored biomaterial interfaces by correlating material chemistry, scaffold architecture, and surface modification parameters with quantitative biological outputs, such as cell adhesion, lineage-specific gene expression, and extracellular matrix deposition [82,83,84,85,86,87]. It also enables inverse design strategies, in which a desired biological outcome, for example, enhanced osteogenic differentiation or controlled macrophage polarization, is used as input to identify the optimal combination of material properties, scaffold geometry, and CAP treatment parameters needed to achieve that response [86]. In practical workflows, AI can be integrated both upstream and downstream of scaffold fabrication and CAP treatment. Machine learning models can predict optimal scaffold architectures and plasma parameters based on target biological outcomes, thereby reducing reliance on empirical trial-and-error approaches. In addition, AI-assisted analysis of imaging and biological datasets provides rapid feedback on scaffold performance, enabling closed-loop optimization that links material design, surface modification, and biological response [82,83,84,85,86]. This approach is particularly advantageous for multifunctional scaffolds incorporating bioactive cues, drug-loaded micro- or nano-reservoirs, or hierarchical architectures.

Moreover, AI-based image analysis (e.g., SEM and confocal microscopy) enhances the characterization of scaffold performance by automating the quantification of fiber orientation, pore interconnectivity, mineralization patterns, ECM deposition, and cell morphology [87]. These tools provide objective and reproducible insights into how CAP-modified and unmodified biomaterials influence tissue regeneration in vitro and in vivo. AI is therefore expected to become an integral component of scaffold design and manufacturing process, including electrospinning, 3D printing, and plasma treatment. ultimately accelerating the development of optimized and patient-specific scaffolds for regenerative medicine.

Despite its considerable potential, the application of AI in biomaterials and TE remains limited by several challenges. Model robustness and generalizability depend on the availability of large, high-quality, and standardized datasets, which are often fragmented or heterogeneous in experimental biomaterials research [82,83,84,85]. In addition, the limited interpretability of complex machine learning models may hinder mechanistic understanding of material–cell interactions, highlighting the need for integrated approaches that combine AI-driven predictions with rigorous experimental validation.

### 3.8. Clinical Translation of Next-Generation Biomaterials

The clinical translation of next-generation biomaterials remains a complex, multifactorial challenge requiring the integration of manufacturing feasibility, regulatory compliance, biological safety, and long-term performance. Despite significant advantages in material design, fabrication technologies, and surface engineering strategies such as CAP, only a limited number of biomaterial-based systems have reached routine clinical use. This translational gap highlights the need to evaluate biomaterials not only for biological efficacy but also for their manufacturability, stability, and compatibility with clinical workflows [22].

Scalability and standardization of manufacturing are primary requirements for clinical translation. Biomaterials intended for human use must be produced under GMP conditions, which is particularly challenging for natural materials and ECM-derived scaffolds due to intrinsic batch-to-batch variability [38,44]. Advanced surface modification strategies (such as CAP treatment, peptide grafting, or nanoparticle functionalization) must also ensure uniformity and stability scale-up. Sterilization represents an additional constraint, as many polymeric and hydrogel-based systems are sensitive to conventional methods [60,70,79]. Therefore, translationally viable biomaterials require robust quality control strategies capable of preserving surface chemistries throughout processing, storage, and distribution.

Comprehensive evaluation of safety and biocompatibility is equally critical. Regulatory frameworks mandate assessment of cytotoxicity, genotoxicity, hemocompatibility, and in vivo responses, including inflammation, fibrosis, and tissue integration. For biodegradable systems, the degradation profile and by-products must be thoroughly characterized [34]. CAP-modified materials introduce additional considerations, such as the temporal stability of surface functional groups, potential aging effects, and residual reactive species (ROS/RNS) [74,75,81]. Demonstrating that CAP treatment selectively modifies only the superficial nanolayer is essential for regulatory approval [76,80].

The interaction between biomaterials and the host immune system is another key determinant of translational success. Early host response, driven by protein adsorption, complement activation, and macrophage polarization, strongly influences long-term outcomes. Materials that promote a pro-regenerative (M2) macrophage phenotype are more likely to support constructive remodeling rather than chronic inflammation. Recent advances in surface nano-engineering, including CAP-treated interfaces, zwitterionic coatings, and ECM-enriched hybrid matrices, have shown promising immunomodulatory effects [22,80].

From a regulatory perspective, classification based on composition and intended use determines the approval pathway. Class I devices are generally limited to low-risk, non-implantable products and are subject to simplified regulatory procedures. Class II devices, such as resorbable scaffolds or certain hydrogels, generally require demonstration of safety and performance through established routes. In contrast, Class III devices (including combination products integrating cells, drugs, or growth factors) require extensive preclinical validation, documentation, and clinical trials [22,82,83]. This complexity further increases for multifunctional systems integrating controlled drug release or bioactive components.

Despite encouraging preclinical results, several barriers continue to hinder clinical translation. Differences between animal models and human physiology limit the predictive value of early studies, particularly for load-bearing applications. Insufficient vascularization remains a major constraint for thick engineered constructs, while long-term remodeling of composite or plasma-modified materials is difficult to predict. These challenges underscore the need for standardized large-animal models and multicenter preclinical studies to improve reproducibility and translational relevance [12,13,36,37].

In addition to scientific and regulatory factors, economic and practical consideration play a decisive role in clinical adoption. Advanced biomaterials (such as ECM-derived scaffolds or plasma-engineered surfaces) are often costly to produce and must exhibit user-friendly handling properties, including flexibility, saturability, and shape retention. Compatibility with existing surgical workflows, together with favorable reimbursement frameworks, significantly influences clinical uptake. Ultimately, only materials that demonstrate clear advantages over current standards are likely to achieve widespread adoption [35,37,58]. Clinically established biomaterials, including collagen membranes, PLGA-based scaffolds, and hyaluronic acid hydrogels, have demonstrated safety and efficacy but remain limited by modest bioactivity and static functionality, highlighting the need for next-generation systems [22,34,37].

Looking ahead, the convergence of personalized manufacturing, surface nano-engineering, and AI-driven predictive tools offers substantial opportunities to enhance the clinical readiness of biomaterials. Patient-specific scaffolds, enabled by 3D printing and advanced imaging, may better match individual anatomical and pathological requirements [62,63]. CAP-based surface engineering provides a versatile means of enhancing bioactivity without altering bulk properties, potentially simplifying regulatory navigation [76,77]. However, CAP-modified biomaterials also pose specific regulatory challenges, including the need to demonstrate surface stability, reproducibility of plasma-induced modifications, and absence of harmful residual reactive species. The lack of standardized CAP protocols remains a key barrier to regulatory approval [22,60,80].

In parallel, AI-driven models can accelerate material optimization by identifying parameters associated with favorable biological responses, reducing reliance on empirical approaches [82,83,85,87].

Collectively, these advances are expected to narrow the gap between laboratory innovation and clinical application, supporting the development of safe, effective, and translationally robust biomaterials for regenerative medicine.

## 4. Conclusions

Recent advances in biomaterials, scaffold fabrication, and surface engineering have markedly enhanced the potential of tissue regeneration strategies. Natural, synthetic, and hybrid materials can now be tailored in terms of composition, mechanics, and architecture. Concurrently, technologies such as electrospinning, 3D/4D printing, and CAP enable precise control over surface properties and cell–material interactions. In parallel, AI is accelerating biomaterial design through predictive, data-driven approaches.

Despite these advantages, the clinical translation of next-generation biomaterials remains challenging. Key issues include manufacturing scalability, batch-to-batch reproducibility, especially for ECM-derived scaffolds, sterilization compatibility, and long-term safety. CAP-modified surfaces and multifunctional composites show considerable promise but require thorough validation of surface stability, immunological effects, and degradation behavior. In addition, more standardized and robust preclinical studies are needed to strengthen regulatory readiness.

Looking forward, the integration of advanced manufacturing, immunomodulatory design, CAP-enabled functionalization, and AI-assisted optimization represents a promising pathway toward clinically translatable biomaterials. The development of scalable, reproducible, and regulatory production pipelines will be essential to bridge the gap between laboratory innovation and clinical application. Continued progress in these areas is expected to enable safer, more effective, and increasingly personalized regenerative therapies.

## Figures and Tables

**Table 4:** List of optimal pore size of various biological tissues.

Tissue	Pore Size (µm)	Function
Skin	100–200 [65]	Promotes fibroblast migration and neovascularization
Bone	200–500 [13]	Enhances osteoblast infiltration and bone formation
Cartilage	250–350 [68]	Supports chondrocyte distribution and ECM deposition
Tendon	20–50 [66]	Guides directional cell growth
Heart (Myocardium)	50–100 [13]	Improves cardiomyocyte viability and oxygen diffusion
Endothelium	30–40 [67]	Limits cellular infiltration during inflammation

Optimal pore size ranges required to modulate cell infiltration, mass transport, and matrix deposition.

**Table 5:** Applications of new findings.

Study	Description	Applications
Percival et al. [23]	Intelligent scaffolds that dynamically respond to biological signals, optimizing the environment for bone growth.	Bone reconstruction; treatment of fractures and bone diseases
Hu et al. [69]	Self-healing hydrogels mimicking the natural extracellular matrix, capable of regenerating after damage.	Implantable medical devices
Ramos et al. [24]	Discovery of lipo-chondrocytes, cells with both adipose and cartilage characteristics.	Cartilage transplants; tissue integration
Crago et al. [60]	Plasma-mediated zwitterionic grafting on polymer surfaces, improving biocompatibility and thrombogenic resistance.	Long-term implantable devices
Gaharwar et al. [22]	Plasma treatment introduces reactive functional groups for anchoring bioactive molecules without altering mechanical properties.	Smart implants; promotion of cell adhesion, proliferation, and differentiation

The new material functionalities translate into targeted clinical or translational applications. These findings provide design principles for next-generation implants, improving tissue regeneration, biocompatibility, and long-term device performance.

## Data Availability

No new data were created or analyzed in this study. Data sharing is not applicable to this article.

## Abbreviations

The following abbreviations are used in this manuscript:

CAP	cold atmospheric plasma
AI	artificial intelligence
TE	tissue engineering
TR	tissue regeneration
ECM	extracellular matrix
ESCs	embryonic stem cells
iPSCs	induced pluripotent stem cells
ADMSCs	adipose-derived mesenchymal stem cells
ADSCs	adipose-derived stem cells
PLA	polylactic acid
PHAs	polyhydroxyalkanoates
HA	hyaluronic acid
PCL	polycaprolactone
PEG	polyethylene glycol
PGA	polyglycolic acid
PLGA	polylactic glycolic acid
AMPs	antimicrobial peptides
miRNAs	microRNAs
MBVs	matrix-bound nanovesicles
SCs	stem/progenitor cells
FGF	fibroblast growth factor
VEGF	vascular endothelial growth factor
PDGF	platelet-derived growth factor
TGF-β	transforming growth factor-beta
FAK	focal adhesion kinase
Src	proto-oncogene tyrosine-protein kinase
PVA/PLLA	poly(vinyl alcohol)/poly(L-lactic acid)
ALP	alkaline phosphatase
MSCs	mesenchymal stem cells
HCOs	human calvaria osteoblasts
GAG	glycosaminoglycan
ROS/RNS	reactive oxygen and nitrogen species
MACI	matrix-induced autologous chondrocyte implantation
